# Posttraumatic stress disorder symptoms among Chinese college students following the COVID-19 outbreak

**DOI:** 10.3389/fnins.2023.1075272

**Published:** 2023-03-07

**Authors:** Jie-Yu Wang, Qi Li, Wei Liu, Yang Yang, Xiao-Guang Wang, Chun-Yan Liu, Xi-Ji Shu, Li Xue, Yan-Wei Shi

**Affiliations:** ^1^Faculty of Forensic Medicine, Zhongshan School of Medicine, Guangdong Provincial Key Laboratory of Brain Function and Disease, Guangdong Province Translational Forensic Medicine Engineering Technology Research Center, Sun Yat-sen University, Guangzhou, China; ^2^Department of Pathology and Pathophysiology, School of Medicine, Wuhan Institute of Biomedical Sciences, Jianghan University, Wuhan, China; ^3^Guangdong Provincial Key Laboratory of Tropical Disease Research, Department of Psychology, School of Public Medicine, Southern Medical University, Guangzhou, China

**Keywords:** COVID-19 pandemic, posttraumatic stress disorder symptoms, risk factors, college students, recommendations

## Abstract

**Objective:**

This study examined the prevalence of posttraumatic stress disorder (PTSD) symptoms in college students 1 month after the lockdown of Wuhan to identify possible risk factors for PTSD symptoms in a cross-sectional study.

**Methods:**

Out of 10,502 who responded, 9,274 students impacted by the COVID-19 pandemic were included in our study. PTSD symptoms was evaluated by the Impact of Event Scale-revised (IES-R). Anxiety/depression symptoms were evaluated by the Kessler Psychological Distress Scale (K10). Personality traits, coping style, and social support were assessed by the Eysenck Personality Questionnaire-Revised Short Scale for Chinese (EPQ-RSC), the Simplified Coping Style Questionnaire (SCSQ), and the Social Support Rating Scale (SSRS). Logistic regression analysis was utilized to further explore risk factors for PTSD symptoms.

**Results:**

More than 1 month after the COVID-19 outbreak, 13.1% of college students developed PTSD symptoms, indicating that COVID-19 associated stressful experiences were connected with PTSD symptoms. After the COVID-19 outbreak, subjects with symptomatologic PTSD symptoms were more likely to experience strained relationships with their family, to have close contact with COVID-19 patients and to drop out of college. The logistic regression model demonstrated the association factors of PTSD symptoms. Neuroticism, psychoticism and an avoidant coping style were associated with increased risk for PTSD symptoms, while an active coping style was protective against PTSD symptoms during this pandemic.

**Conclusion:**

The results showed that PTSD symptoms was prevalent in Chinese college students 1 month after the COVID-19 outbreak. Effective psychological support work should be carried out accordingly.

## Introduction

The coronavirus disease 2019 (COVID-19) is a new infectious disease that has swept the world more than 2 years. According to reports from the World Health Organization (WHO), there have been 620,878,405 confirmed cases globally by 14 October, 2022 including 6,543,138 death (WHO, 2022). To prevent virus transmission, the Chinese government adopted unprecedented, extreme measures to mitigate the outbreak, such as nationwide or regional lockdowns were linked with increased stressful events and limited social interactions. This situation has also triggered a wide variety of psychological problems, such as anxiety, depression and posttraumatic stress disorder (PTSD) (Xiong et al., 2020).

Posttraumatic stress disorder is a disorder that develops in some people who have experienced a shocking, scary, or dangerous event. Based on Life Events Checklist for DSM-5 (Sun et al., 2021), being involved in a life-threatening illness might be a traumatic experience. Findings from recent studies have shown that COVID-19 can be identified as a traumatic stressor and can cause PTSD symptoms in the general public (Forte et al., 2020). A meta-analysis of 14 studies conducted during the first wave of the pandemic, between February and April, revealed a high rate of COVID-19 related PTSD (23.88%) in the general population (Cooke et al., 2020). A nationwide survey in China with more than 50 thousand participants found that the prevalence was 35% (Qiu et al., 2020).

China has around 33 million students studying on college campuses. The psychological impact of the new COVID-19 is a public health concern (Tang et al., 2020). College students are particularly susceptible to mental health deterioration during the ongoing pandemic. Chinese college students have been exposed to a significant number of COVID-19-related stressful events during the outbreak, including disruptions in their academic studies, entertainment, and family and social lives. But the prevalence rate of PTSD in college students from different regions presents a wide range of variety. In the group of home-quarantined Chinese university students in Chengdu (*n* = 2,485) 1 month after the breakout, the prevalence was 2.7% (Tang et al., 2020). However, Chi et al. (2020) revealed that in a sample of Chinese students distributed across 29 provinces and cities of China (*n* = 2,038), the prevalence of clinically relevant PTSD reached 30.8% during the pandemic. In other parts of the world, the rate of probable PTSD 1 month after the COVID-19 lockdown was 19.5% among a large sample of French university students (*n* = 22,883) (Wathelet et al., 2021), one quarter (25.4%) of American medical students, 29.0% in Asian medical students and 51.0% in Europe medical students (Peng et al., 2022). It has been demonstrated that population in different regions may have different prevalence rates of PTSD.

Posttraumatic stress disorder symptom is a core criterion for the diagnosis of PTSD, which could last for at least 1 month. Most people can recover from initial symptoms naturally, while others’ symptoms may worsen, lasting for months or even years and interfering with daily life functioning. Previous studies, the samples from Chengdu and 29 provinces and cities of China, were conducted 1 month after the breakout (Tang et al., 2020). However, they were not focused on the population mainly in Hubei province most affected by the COVID-19 outbreak in the initial wave of the pandemic.

This study aimed to evaluate the prevalence and risk factors of PTSD symptoms in the college students’ data. To meet PTSD duration criteria, data were mainly collected in Hubei province more than 1 month after the peak outbreak. The main findings of this study would offer useful early detection and intervention methods to PTSD, and provide helpful insight into planning for future outbreaks of emerging infectious diseases.

## Materials and methods

### Study design and participants

The study used a cross-sectional survey on 15–24 March 2020, 1 month after the lockdown of Wuhan. We’ve designed the questionnaire *via* the WeChat-based survey program Questionnaire Star. Our online questionnaire was sent out to different student WeChat groups in many universities. By convenient sampling, participants were selected to receive a link to the online survey platform (WeChat) where they could fill in the questionnaire at their own time. They were required to fill in the name of their universities. The exact number of universities was counted at the end of data collection. Our collected samples were from different grades and majors in 72 universities, covering a wide range of disciplines, including science, medicine, engineering, literature and so on. Among these 72 universities, about 99.6% samples came from universities of Hubei province.

A scripted set of instructions was used to introduce the questionnaires, including a statement that there were no right or wrong answers to the questions being asked. The purpose of instructions was to identify how the participants felt about or experienced the topic under investigation. The consent form and all study procedures were approved by the Human Subjects Committee at Zhongshan School of Medicine, Sun Yat-sen University. Written informed consent was obtained from all subjects before the study.

### Survey instruments

The questionnaire covers sociodemographic characteristics, PTSD symptomology, COVID-19-related stressful events, and other issues. Variables included personality traits, coping strategies and social support. These variables, as moderators between stressors and psychological responses, were also evaluated. The demographic information of the participants included sex, age, living area, marital status, grade, annual family income and geographic region (Wuhan or out of Wuhan).

Symptoms of PTSD were measured by the 22-item self-administered Impact of Event Scale-revised (IES-R) (Creamer et al., 2003), scored from 0 to 88. It has demonstrated reliability and validity to measure PTSD symptoms across different cultures and settings. We assessed respondents who met “probable diagnosis” of PTSD using an inputted 1.5 mean item cut-off score, equivalent to a 33/88 total score on the IES-R (Creamer et al., 2003). The sum of the scores of Intrusion subscale and avoidance subscale could demonstrate the order of severity of PTSD symptoms: 0–8 points means subclinical PTSD symptoms; 9–25 points means mild PTSD symptoms; 26–43 points means moderate PTSD symptoms: above 44 points means severity PTSD symptoms.

Next, the Kessler Psychological Distress Scale (K10) was used to measure non-specific psychological distress. It has been shown to be a sensitive screening tool for the Diagnostic and Statistical Manual of Mental Disorders (DSM-IV) criteria for anxiety and mood disorders. Responses are given on a 5-point Likert-type scale ranging from 1 (none of the time) to 5 (all of the time) (Easton et al., 2017). Response scores are summed to create a total score (range = 10–50), with higher scores signifying more psychological distress. We assessed participants’ experience of COVID-19-related stressful events (stressors), and developed a checklist. These events were grouped into four categories: (a) family-/friend-related events (4 items); (b) self-related events (8 items); (c) information-related events (1 item); (d) COVID-19-related stressful events (6 items). Stressful events prior to COVID-19 were also investigated (5 items). Participants answered whether they experienced these stressful events during the past month. Personality traits were measured by the Eysenck Personality Questionnaire-Revised Short Scale for Chinese (EPQ-RSC) (Eysenck et al., 1985; Qian et al., 2000). This questionnaire includes four aspects: extraversion (E), neuroticism (N), psychoticism (P) and a lie detector inventory. The Simplified Coping Style Questionnaire (SCSQ) (Xie, 1985) was used also to test two types of coping style. Positive stress-coping strategies include problem solving, help seeking, and reconstruction, while negative stress-coping strategies include avoidance and distraction (Yang et al., 2014; Sun et al., 2019). The Social Support Rating Scale (SSRS) is specifically designed to evaluate (Cheng et al., 2008; Edward et al., 2020) subjective support, objective support and utilization of support. The total score on the SSRS ranges from 12 to 66, with higher scores indicating better perceived social support.

### Statistical analysis

The data were analyzed using SPSS V.19. Statistical significance was defined as a two-tailed *p*-value less than 0.05. The original scores of the measurement tools were not normally distributed and are presented as medians with interquartile ranges (IQRs). Frequencies, proportions, 95% CIs of proportions, and Chi-squared tests were generated to examine the relationships between demographic variables (gender, age, grade, marital status, residence, annual family income, and locations during the epidemic) and the development of PTSD symptoms. The non-parametric Mann–Whitney U test and Kruskal–Wallis test were applied to compare the scores of mediating factors of PTSD between the two groups. To determine potential risk factors for developing PTSD, binary logistic regression analysis was performed. The associations between risk factors and outcomes are presented as odds ratios (ORs) and 95% CIs, including sex, age, grade, marital status, residence, annual family income and locations during the pandemic.

## Results

### Development of PTSD symptoms among Chinese college students

In our survey, all the answers must be fully filled before submission. We received a total of 10,502 responses. Of these, we excluded 1,228 with extremely short answer times or the same answers given throughout the questionnaire or scales. Thus, 9,274 students from eight universities comprised the valid sample in our study.

According to the cut-off for “probable diagnosis” of PTSD in the IES-R, all respondents were divided into two groups: the PTSD symptoms positive group and the PTSD symptoms negative group. There were 1,211 (13.1%) students who met the levels of probable PTSD diagnosis. The scores of three items of IES-R were listed in Table 1. According to the IES-R, sum of the scores of Intrusion subscale and avoidance subscale could demonstrate the order of severity of PTSD symptoms. There were 4,380 (47.23%) individuals with subclinical PTSD symptoms and 121 (1.3%) with the severe PTSD symptoms.

**TABLE 1:** Percentage of the different order of severity of PTSD symptoms.

PTSD symptoms severity	Number (%)
Subclinical	4,380 (47.23)
Mild	3,719 (40.10)
Moderate	1,054 (11.37)
Severe	121 (1.30)

**FIGURE 1:**
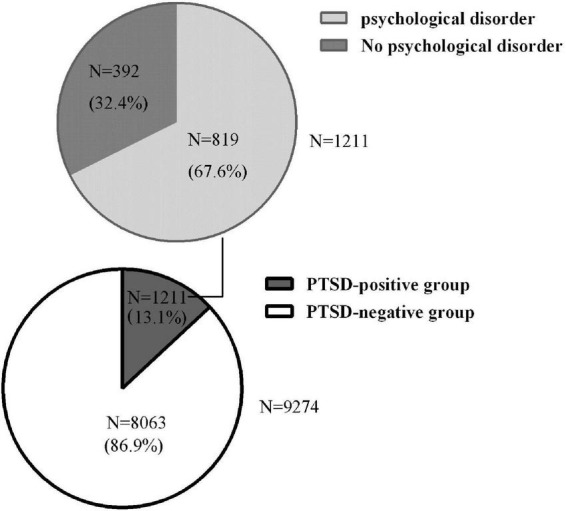
Percentage of COVID-19-associated PTSD symptoms, anxiety, and depressive symptoms co-existing within the PTSD sample.

Among the students met the levels of probable PTSD diagnosis, 819 (67.6%) students met the cut-off of K10 for psychological disorder (Figure 1). Compared with the PTSD symptoms negative group, the PTSD symptoms positive group reported significantly higher scores on symptoms of depression and anxiety [median (IQR) 17.00 (13.00–19.00) vs. 8.00 (6.00–12.00), *P* < 0.001; median (IQR) 11.00 (9.00–13.00) vs. 5.00 (4.00–8.00), *P* < 0.001] (Table 2).

**TABLE 2:** Comparison of anxiety and depression scores between the PTSD symptoms positive group and PTSD symptoms negative group.

	K10 total score	Z	*P*	K10-anxiety	Z	*P*	K10-depression	Z	*P*
	**Median (IQR)**			**Median (IQR)**			**Median (IQR)**		
PTSD-positive group (*N* = 1,211, 13.1%)	28.00 (22.0–32.00)	−44.15	<0.001	11.00 (9.00–13.00)	−44.06	<0.001	17.00 (13.00 –19.00)	–42.86	<0.001
PTSD-negative group (*N* = 8,063, 86.9%)	13.00 (10.00–190.0)			5.00 (4.00–8.00)			8.00 (6.00–12.00)		

### Correlation between the development of COVID-19-related PTSD symptoms and demographic characteristics

The demographic and selected characteristics of the study population are shown in Table 3. Within the valid sample, a majority of participants were female [5,574 (60.10%)], were aged 18–20 years [5,322 (57.39%)] and were single without a lover or partner [7,417 (79.98%)]. The family annual income of a total of 4,057 participants (43.75%) was less than 50,000 RMB. Approximately half of students lived in urban and half lived in rural areas [4,633 (49.96%) vs. 4,641 (50.04%)].

**TABLE 3:** Differences in demographic variables between the PTSD symptoms positive group and PTSD symptoms negative group.

Variable		Total no. (%)	PTSD-positive group no. (%)	PTSD-negative group no. (%)	χ^2^	95% CI	*P*
Sex	Male	3,700 (39.90)	445 (12.03)	3,255 (87.97)	5.764	(10.98%, 13.08%)	0.016
	Female	5,574 (60.10)	766 (13.74)	4,808 (86.26)		(12.84%, 14.65%)	
Age	<18	60 (0.64)	5 (8.33)	55 (91.67)	3.799	(1.13%, 15.53%)	0.284
	18–20	5,322 (57.39)	671 (12.61)	4,651 (87.39)		(11.72%, 13.5%)	
	21–23	3,717 (40.08)	510 (13.72)	3,207 (86.28)		(12.61%, 14.83%)	
	≥24	175 (1.89)	25 (14.29)	150 (85.71)		(9.05%, 19.52%)	
Marital status	Single	7,417 (79.98)	930 (12.54)	6,487 (87.46)	8.797	(11.77%, 13.29%)	0.003
	Not single	1,857 (20.02)	281 (15.13)	1,576 (84.87)		(13.45%, 16.64%)	
Place of residence	Urban	4,633 (49.96)	588 (12.69)	4,045 (87.31)	1.095	(11.73%, 13.65%)	0.309
	Rural	4,641 (50.04)	623 (13.42)	4,018 (86.58)		(12.44%, 14.40%)	
Annual family income	<50,000 RMB	4,057 (43.75)	591 (14.57)a*	3,466 (85.43)a	16.802	(13.48%, 15.65%)	0.001
	50,000–100,000 RMB	2,992 (32.26)	366 (12.23)b	2,626 (87.77)b		(11.06%, 13.41%)	
	100,000–200,000 RMB	1,628 (17.55)	177 (10.87)b	1,451 (89.13)b		(9.36%, 12.39%)	
	>200,000 RMB	597 (6.44)	77 (12.90)a,b	520 (87.10)a,b		(10.20%, 15.59%)	
Grade	Junior (1st or 2nd year)	5,151 (55.54)	630 (12.23)	4,521 (87.77)	6.986	(13.03%, 15.15%)	0.008
	Senior (≥ 3rd year)	4,123 (44.56)	581 (14.09)	3,542 (85.91)		(11.34%, 13.13%)	
Location	Wuhan	3,321 (35.81)	457 (13.76)	2,864 (86.24)	2.252	(12.59%, 14.93%)	0.133
	Not Wuhan	5,953 (64.19)	754 (12.67)	5,199 (87.33)		(11.82%, 13.51%)	

*Means in a line followed by the same letters are not significantly different (*p* > 0.05).

According to the cut-off for “probable diagnosis” of PTSD in the IES-R, all respondents were divided into two groups: the PTSD symptoms positive group and the PTSD symptoms negative group. There were 1,211 (13.1%) students who met the levels of probable PTSD diagnosis. Based on the cut-off of the IES-R, students with PTSD-positive symptoms included a significantly greater percentage of female than male students [766 (13.74%) vs. 445 (12.03%); *P* < 0.001], significantly more not-single students than single students [281 (15.13%) vs. 930 (12.54%); *P* < 0.001], significantly less students with an annual family income less than 50,000 than students with an annual family income more than 50,000 [591 (14.57%) vs. 620 (36.00%); *P* = 0.001], and significantly more senior students than junior students [581 (14.09%) vs. 630 (12.23%); *P* = 0.008] (Table 1). Although the differences did not reach statistical significance, the PTSD symptoms positive group included more students from rural areas, older than 24 years and living in Wuhan.

### Correlation between the development of COVID-19-related PTSD symptoms and stressful events

Table 4 describes respondents’ experiences with COVID-19 and associations with PTSD symptoms, which are ranked by OR value in descending order. For COVID-19-related stressful events, students who experienced strained relationships with family or others were more likely to report PTSD symptoms (OR = 3.51, 95% CI 2.99–4.13, *P* < 0.001) than those who did not. Students who dropped out, another COVID-19-related stressful event, had a threefold risk of developing PTSD symptoms (OR = 3.04, 95% CI 2.42–3.82, *P* < 0.001). Students with trauma-related difficulties related to economic status, changed life patterns and high study pressure were more likely to report PTSD symptoms than those who did not have these experiences (OR = 2.24, 95% CI 1.98–2.53, *P* < 0.001; OR = 2.73, 95% CI 2.41–3.09, *P* < 0.001; OR = 2.92, 95% CI 2.58–3.31, *P* < 0.001). For self-related events, respondents who knew at least one person who died from COVID-19 or knew someone quarantined due to COVID-19 exposure were also more likely to report PTSD symptoms (OR = 2.95, 95% CI 2.13–4.10, *P* < 0.001; OR = 2.00, 95% CI 1.76–2.28, *P* < 0.001). For family-/friend-related events, respondents who experienced the death of a family member or relative or suffered a major family crisis faced higher risk of PTSD (OR = 2.95, 95% CI 2.13–4.10, *P* < 0.001; OR = 2.55, 95% CI 1.69–3.84, *P* < 0.001) than those who did not. For information-related events, students who were exposed to internet information were more likely to experience PTSD symptoms (OR = 2.35, 95% CI 1.83–3.02, *P* < 0.001) (Table 3) than those who were not.

**TABLE 4:** Correlation between the development of COVID-19-related PTSD symptoms and stressful events.

	No. (%) of yes responses	PTSD-positive group no. (%)	PTSD-negative group no. (%)	χ^2^	OR (95% Cl)	*P*
**Stressful events during pandemic**						
Your relationship with your family has been strained during the epidemic.				261.42	3.51 (2.99–4.13)	< 0.001
Yes	853 (9.20)	263 (30.83)	590 (69.17)			
No	8,421 (90.80)	948 (11.26)	7,473 (88.74)			
You had close contact with COVID-19 patients.				35.98	3.15 (2.12–4.67)	<0.001
Yes	117 (1.26)	37 (31.62)	80 (68.38)			
No	9,157 (98.74)	1,174 (12.82)	7,983 (87.18)			
You are waiting to drop out during the epidemic.				99.04	3.04 (2.42–3.82)	<0.001
Yes	377 (4.07)	113 (29.97)	264 (70.03)			
No	8,897 (95.93)	1,098 (12.34)	7,799 (87.66)			
One of your family members or friends died of COVID-19.				45.97	2.95 (2.13–4.10)	<0.001
Yes	176 (1.90)	53 (30.11)	123 (69.89)			
No	9,098 (98.10)	1,158 (12.73)	7,940 (87.27)			
Your work/study pressure during the epidemic is very high.				306.73	2.92 (2.58–3.31)	<0.001
Yes	3,442 (37.11)	724 (21.03)	2,718 (78.97)			
No	5,832 (62.89)	487 (8.35)	5,345 (91.65)			
Your life pattern has changed significantly during the epidemic.				264.07	2.73 (2.41–3.09)	<0.001
Yes	3,768 (40.63)	751 (19.93)	3,017 (80.07)			
No	5,506 (59.37)	460 (8.35)	5,046 (91.65)			
One of your family members or friends has suffered serious injuries, natural disasters or other crises.				21.32	2.55 (1.69–3.84)	<0.001
Yes	117 (1.26)	32 (27.35)	85 (72.65)			
No	9,157 (98.74)	1,179 (12.88)	7,978 (87.12)			
You have met confirmed/suspected COVID-19 patients.				24.85	2.50 (1.72–3.62)	<0.001
Yes	145 (1.56)	39 (26.90)	106 (73.10)			
No	9,129 (98.44)	1,172 (12.84)	7,957 (87.16)			
You get a lot of epidemic information from your mobile phone/the Internet every day.				46.68	2.35 (1.83–3.02)	<0.001
Yes	8,203 (88.45)	1,142 (13.92)	7,061 (86.08)			
No	1,071 (11.55)	69 (6.44)	1,002 (93.56)			
You have experienced financial difficulties during the epidemic.				172.97	2.24 (1.98–2.53)	<0.001
Yes	3,347 (36.09)	642 (19.18)	2,705 (80.82)			
No	5,927 (63.91)	569 (9.60)	5,358 (90.40)			
You have experienced fever, cough, and other COVID-19 symptoms during the epidemic.				59.99	2.22 (1.81–2.73)	<0.001
Yes	549 (5.92)	131 (23.86)	418 (76.14)			
No	8,725 (94.08)	1,080 (12.38)	7,645 (87.62)			
You are a confirmed/suspected COVID-19 patient.				10.25	2.21 (1.34–3.62)	0.001
Yes	85 (0.92)	21 (24.71)	64 (75.29)			
No	9,189 (99.08)	1,190 (12.95)	7,999 (87.05)			
Your relatives and friends have been forced to stay at home or in a designated place of isolation and observation.				115.02	2.00 (1.76–2.28)	<0.001
Yes	2,198 (23.70)	435 (19.79)	1,763 (80.21)			
No	7,076 (76.30)	776 (10.97)	6,300 (89.03)			
You may have met confirmed/suspected COVID-19 patients.				72.26	2.00 (1.70–2.36)	<0.001
Yes	1,035 (11.16)	222 (21.45)	813 (78.55)			
No	8,239 (88.84)	989 (12.00)	7,250 (88.00)			
You cannot be reunited with your family (lover) during the epidemic.				68.74	1.81 (1.57–2.08)	<0.001
Yes	1,622 (17.49)	314 (19.36)	1,308 (80.64)			
No	7,652 (82.51)	897 (11.72)	6,755 (88.28)			
You have a family member or friend with confirmed/suspected COVID-19.				33.01	1.80 (1.47–2.21)	<0.001
Yes	635 (6.85)	130 (20.47)	505 (79.53)			
No	8,639 (93.15)	1,081 (12.51)	7,558 (87.49)			
You are at risk of getting infected because of your work during the epidemic.				8.21	1.71 (1.18–2.48)	0.004
Yes	178 (1.92)	36 (20.22)	142 (79.78)			
No	9,096 (98.08)	1,175 (12.92)	7,921 (87.08)			
Your work during the epidemic is related to fighting the epidemic.				16.16	1.66 (1.30–2.14)	<0.001
Yes	414 (4.46)	81 (19.57)	333 (80.43)			
No	8,860 (95.54)	1,130 (12.75)	7,730 (87.25)			
You have been forced to stay at home or in a designated place of isolation and observation.				61.08	1.65 (1.45–1.87)	<0.001
Yes	2,611 (28.15)	455 (17.43)	2,156 (82.57)			
No	6,663 (71.85)	756 (11.35)	5,907 (88.65)			
**Stressful events prior to pandemic**						
Your relationship with your family was strained before the epidemic.				135.91	3.76 (2.96–4.76)	<0.001
Yes	325 (3.50)	112 (34.46)	213 (65.54)			
No	8,949 (96.50)	1,099 (12.28)	7,850 (87.72)			
You were waiting for employment or facing unemployment before the epidemic.				20.97	3.09 (1.86–5.14)	<0.001
Yes	70 (0.75)	22 (31.43)	48 (68.57)			
No	9,204 (99.25)	1,189 (12.92)	8,015 (87.08)			
You were under great pressure to work/study before the epidemic.				255.41	2.72 (2.40–3.08)	<0.001
Yes	2,219 (23.93)	511 (23.03)	1,705 (76.97)			
No	7,055 (76.07)	700 (9.92)	6,355 (90.08)			
You suffered a major family crisis (death, serious illness, serious injuries, or financial bankruptcy) before the epidemic.				28.20	1.85 (1.47–2.33)	<0.001
Yes	469 (5.06)	99 (21.11)	370 (78.89)			
No	8,805 (94.94)	1,112 (12.63)	7,693 (87.37)			
You were seriously ill or seriously injured before the epidemic.				2.81	1.81 (0.90–3.64)	0.094
Yes	47 (0.51)	10 (21.28)	37 (78.72)			
No	9,227 (99.49)	1,201 (13.02)	8,026 (86.98)			

### Factors associated with COVID-19-related PTSD symptoms

**FIGURE 2:**
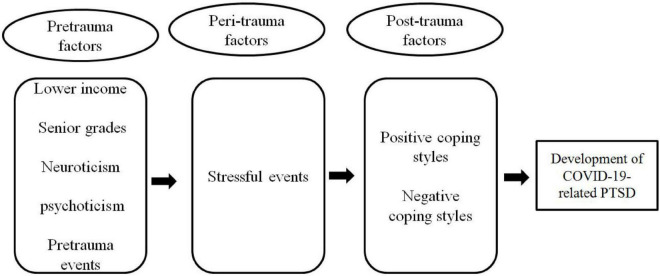
Factors related to COVID-19-associated PTSD symptoms among Chinese college students.

The findings of the binary logistic regression model assessing risk factors for PTSD morbidity are displayed in Table 5. Senior grade [OR = 1.18 (95% CI, 1.03–1.34), *P* = 0.016], annual family income (ref = less than 50,000, *P* = 0.004), neuroticism [OR = 1.07 (95% CI, 1.06–1.07), *P* < 0.001], psychoticism [OR = 1.01 (95% CI, 1.01–1.02), *P* = 0.001], positive coping styles [OR = 0.970 (95% CI, 0.96–0.98), *P* < 0.001], and negative coping styles [OR = 1.15 (95% CI, 1.13–1.17), *P* < 0.001] were significantly related to PTSD symptoms. Variables such as gender, age, marital status, place of residence, location, and social support were dropped from this regression model. Among these risk factors, grade, annual family income, neuroticism and psychoticism are pretrauma factors, while positive coping styles and negative coping styles are posttraumatic factors. It was suggested that the COVID-19-related PTSD symptoms are the results of multiple factors (Figure 2).

**TABLE 5:** Logistic regression model of PTSD symptoms development among Chinese college students^a^.

Factors	Correlation coefficient	Adjusted OR (95% CI)	*P*
**Grade**			
[Senior = 1; Junior = 0 (ref)]	0.16	1.18 (1.03–1.34)	0.016
**Annual family income**			
[less than RMB 50,000 = 1 (ref)]			0.004
Between RMB 50,000 and RMB 100,000	-0.20	0.82	0.012
Between RMB 100,000 and RMB 200,000	-0.32	0.73	0.001
More than RMB 200,000	-0.08	0.92	0.580
**Coping style**			
Positive coping style	-0.03	0.97 (0.96–0.98)	<0.001
Negative coping style	0.14	1.15 (1.13–1.17)	<0.001
**Personality traits**			
Psychoticism	0.01	1.01 (1.01–1.02)	0.001
Neuroticism	0.07	1.07 (1.06–1.07)	<0.001

^a^Factors entered into stepwise forward binary logistic regression include gender, age, grade, annual family income, place of residence, location during epidemic, psychoticism, neuroticism, extraversion, positive coping style, negative coping style, subjective support, objective support, and utilization of support.

## Discussion

This study, which was conducted more than 1 month after the COVID-19 high-incidence period, focused on the PTSD symptoms prevalence and risk factors among college students in China. The prevalence of PTSD symptoms in Chinese public 1 month after the COVID-19 outbreak was 4.6% (Sun et al., 2021). Our study showed a higher prevalence 13.1%. It mainly resulted from the samples in our study mainly come from Hubei province, the original and worst disaster-affected area. In fact, the pandemic is associated with other mental health symptoms and disorders. A meta-analysis has shown that population have mental health problems such as anxiety, depression, insomnia, PTSD and so on caused by COVID-19 outbreak, prevalence of which in populations affected by COVID-19 is much more times than in general population. Even without infection, many people believed that this unknown new virus was highly contagious that could cause death or disability. Unknown transmission routes of virus and uncertainty treatment made them feel fear, worry, and helpless. In addition, financial pressure triggered by the pandemic like job losses, wage losses, and uncertainty about the future (Nicola et al., 2020) also effected mental health.

Several studies have shown the prevalence of PTSD among college students in response to COVID-19. Around the world, PTSD prevalence rates in American, Asian, and Europe medical students are higher than that in our study. It may be because of effective and strict control of the epidemic in China (Peng et al., 2022). In China, from six universities of two large cities in southwest China, the PTSD prevalence was 2.7% in a sample of home-quarantined students (Tang et al., 2020), much lower than our results. This may because the samples in our study mainly come from Hubei province, the original and worst disaster-affected area. In addition, these inconsistent data regarding the prevalence of PTSD in the student population may also result from using different measurement. Previous studies used the PCL-5 (Cutoff Score 32) (Sun et al., 2021), or PCL-C (Tang et al., 2020), while IES-R was used in our study.

The prevalence in our study was lower than that of earthquake-associated PTSD (from 15.6 to 24.2%) (Wang et al., 2000; Xi et al., 2017) and flood-associated PTSD (15.4%) (Zhou et al., 2013) reported in studies conducted 6 months, 9 months, or more than 10 years after disaster outbreaks, much longer than the 1-month period in our study. Previous research on the mental health during infectious disease outbreaks. Medical staff involved in the 2003 severe acute respiratory syndrome (SARS) outbreak found that about 10% of the sample reported high levels of PTSD symptoms (Wu et al., 2009). Another study showed that about 2% Chinese university students met symptom criteria for PTSD during 2009 H1N1 pandemic (Xu et al., 2011). Differences in the prevalence of PTSD symptoms may be attributed to differences in the intensity and type of trauma exposure. Although the psychological impact of disasters may evolve over time (Hu et al., 2015), earthquakes and floods are often much more devastating and destructive than infectious diseases and occur unexpectedly. The infectiousness could be seen everywhere, but people cannot find where the real threats come from. People could not realize where the source of the infection is, so they need to prevent infectious diseases everywhere, which could become chronic and unpredictable stressors (d’Ettorre et al., 2021).

A high probability of PTSD symptoms development was found among college students who had strained relationships with family (OR = 3.5). During the COVID-19 epidemic, college students were instructed not to go out, meet people, organize or attend mass gatherings. Family members seemed to be the first choice to provide any kind of social support. Our results imply that improving relationship with family may decrease the incidence of PTSD symptoms. Other event exposure factors were ranked according to risk intensity as follows: close contact with COVID-19 patients, dropping out, death of a relative or friend from COVID-19, high study pressure, changed life pattern, financial difficulties and any quarantining. It is suggested that among college students, a wide range of stressors occur concurrently during traumatic exposure and are often causally related, which the features of stressful events among college students are.

Some demographic factors may also influence PTSD symptoms development during the COVID-19 pandemic. Senior student and lower income was identified as two risk factors. These may be attributed to the fact that senior students face to pressure of employment after graduation, academic pressure and economic hardship.

Positive coping style and negative coping style were all entered the regression model. Active coping involves focusing on the cause of the stress and attempting to do something actively in order to reduce the stress. Individuals often use avoidant coping when they wish to reduce the emotional stress elicited by a problematic situation rather than deal with the stress at its source (Carver et al., 1989). Previous studies have demonstrated that coping style is depended upon the controllability of stressors (Main et al., 2011). For example, coping with SARS-related stressors, college students reported avoidant coping may be more adaptive than active coping (Main et al., 2011). Like SARS-related stressors, COVID-19-related stressors may also be regarded as uncontrollable stressors. It is speculated that avoidant coping may be more adaptive for college students during COVID-19 pandemic. Further studies could be focused on the relations between coping and individuals’ psychological adjustment during COVID-19 outbreak.

This study also showed that neuroticism and psychoticism were two significant predictors in the personality dimension. This finding of neuroticism in the context of the pandemic is in line with previous findings in various contexts, such as fights, transportation accidents, industrial and domestic accidents, terrorism, violent crimes and explosions (Liang et al., 2019). The relationship between neuroticism and stress might be due to a low ability of individuals with high neuroticism scores to cope effectively with stressors, which would increase the amount of stress (Yin et al., 2019). Similar to our results, research has found a strong correlation between traffic accident-related PTSD and flood-related PTSD and psychoticism. Overall, negative emotions, including neuroticism, avoidance, hostility, anger, and anxiety, are likely to predict PTSD (Su et al., 2018).

The following recommendations for psychological prevention and interventions were proposed according to our present findings:

(1) According to the intangible and long-lasting stress characteristics of a pandemic, different mental health interventions need to be developed at different periods of the outbreak (i.e., before, during, and after the outbreak).
(2) At the early stage of epidemic, it is necessary to conduct a rapid assessment of the mental health, including family and cultural background, of college students through the cooperation between psychological agencies and universities. This ensures that individuals with neurotic and psychotic personalities, negative coping strategies or family tensions receive more professional support.
(3) During quarantine, online psychological service established by collaboration between psychological agencies (e.g., Psychological First Aid), government and universities, should serve as an effective tool to support college students. Focusing on helping students avoid negative coping strategies and cultivate optimistic thinking styles.
(4) The construction of the cooperation platform between the University and the government can ensure that update and accurate information about employment is readily available and accessible to senior students. Information should include how to seek job, as well as messages to promote psychological wellbeing during or after graduation.
(5) We should establish a relief system for low-income students from universities and local governments. Schools should regularly and supportively monitor low income students and ensure good quality mental and material help are provided to them.

### Limitations

The results of this study should be considered with several potential limitations. First, the prevalence of PTSD in this study was estimated by an online questionnaire rather than a clinical interview. Secondly, this study is a cross-sectional study and no causal conclusion can be drawn. In addition, although our samples came from 72 universities, most of our samples came from only one university in Wuhan of HuBei province.

## Conclusion

The strengths of this study include its large sample size and the special study period. The results of this survey indicate that PTSD symptoms may have been relatively high incidence among Chinese college students, especially among senior students with lower income. Strained relationship with family was the most probably associated with PTSD. The findings identify populations of college students at risk for PTSD symptoms during the COVID-19 pandemic and may help in implementing mental health intervention policies in other countries and regions.

## Data availability statement

The raw data supporting the conclusions of this article will be made available by the authors, without undue reservation.

## Ethics statement

The studies involving human participants were reviewed and approved by the Human Subjects Committee at Zhongshan School of Medicine, Sun Yat-sen University. The patients/participants provided their written informed consent to participate in this study.

## Author contributions

J-YW and QL collected and analyzed the data and wrote the manuscript. WL, YY, X-GW, C-YL, and X-JS collected the data. Y-WS and LX conceived, designed, and organized the study in addition to interpreting the results and revising the manuscript. All authors read and approved the final manuscript.

## References

[B1] Carver C. S. Scheier M. F. Weintraub J. K. (1989). Assessing coping strategies: a theoretically based approach. *J. Pers. Soc. Psychol.* 56 267–283. 10.1037/0022-3514.56.2.267 2926629

[B2] Cheng Y. Liu C. Mao C. Qian J. Liu K. Ke G. (2008). Social support plays a role in depression in Parkinson’s disease: a cross-section study in a Chinese cohort. *Park Relat. Disord.* 14 43–45. 10.1016/j.parkreldis.2007.05.011 17616476

[B3] Chi X. Becker B. Yu Q. Willeit P. Jiao C. Huang L. (2020). Prevalence and psychosocial correlates of mental health outcomes among Chinese college students during the coronavirus disease (COVID-19) pandemic. *Front. Psychiatry* 11:803. 10.3389/fpsyt.2020.00803 32848958 PMC7427603

[B4] Cooke J. E. Eirich R. Racine N. Madigan S. (2020). Prevalence of posttraumatic and general psychological stress during COVID-19: a rapid review and meta-analysis. *Psychiatry Res.* 292:113347. 10.1016/j.psychres.2020.113347 PMC7392847 32763477

[B5] Creamer M. Bell R. Failla S. (2003). Psychometric properties of the impact of event scale-revised. *Behav. Res. Ther.* 41 1489–1496. 14705607 10.1016/j.brat.2003.07.010

[B6] d’Ettorre G. Ceccarelli G. Santinelli L. Vassalini P. Innocenti G. P. Alessandri F. (2021). Post-traumatic stress symptoms in healthcare workers dealing with the COVID-19 pandemic: a systematic review. *Int. J. Environ. Res. Public Health* 18:601. 10.3390/ijerph18020601 33445712 PMC7828167

[B7] Easton S. D. Safadi N. S. Wang Y. Hasson R. G. (2017). The kessler psychological distress scale: translation and validation of an Arabic version. *Health Qual. Life Out.* 15:215. 10.1186/s12955-017-0783-9 PMC5658946 29078774

[B8] Edward W. W. C. Wen C. Ip I. C. N. Hall B. J. (2020). Effects of social support and depression on problematic drinking among trauma-exposed chinese adults: a population-based study. *Heliyon* 6:e03405. 10.1016/j.heliyon.2020.e03405 32099926 PMC7031303

[B9] Eysenck S. B. G. Eysenck H. J. Barrett P. (1985). A revised version of the psychoticism scale. *Per. Individ. Differ.* 6 21–29.

[B10] Forte G. Favieri F. Tambelli R. Casagrande M. (2020). COVID-19 pandemic in the Italian population: validation of a post-traumatic stress disorder questionnaire and prevalence of PTSD symptomatology. *Int. J. Environ. Res. Public Health* 17:4151. 10.3390/ijerph17114151 32532077 PMC7312976

[B11] Hu S. Tan H. Cofie R. Zhou J. Yang T. Tang X. (2015). Recovery from posttraumatic stress disorder after a flood in China: a 13-year follow-up and its prediction by degree of collective action. *BMC Public Health.* 15:615. 10.1186/s12889-015-2009-6 26148510 PMC4491893

[B12] Liang Y. Cheng J. Ruzek J. I. Liu Z. (2019). Posttraumatic stress disorder following the 2008 Wenchuan earthquake: a 10-year systematic review among highly exposed populations in China. *J. Affect. Disord.* 243 327–339. 10.1016/j.jad.2018.09.047 30261448

[B13] Main A. Zhou Q. Ma Y. Luecken L. J. Liu X. (2011). Relations of sars-related stressors and coping to Chinese college students’ psychological adjustment during the 2003 Beijing SARS epidemic. *J. Couns. Psychol.* 58 410–423. 10.1037/a0023632 21574694

[B14] Nicola M. Alsafi Z. Sohrabi C. Kerwan A. Al-Jabir A. Iosifidis C. (2020). The socio-economic implications of the coronavirus and COVID-19 pandemic: a review. *Int. J. Surg.* 78 185–193. 32305533 10.1016/j.ijsu.2020.04.018 PMC7162753

[B15] Peng P. Hao Y. Liu Y. Chen S. Wang Y. Yang Q. (2022). The prevalence and risk factors of mental problems in medical students during COVID-19 pandemic: a systematic review and meta-analysis. *J. Affect. Disord.* 321 167–181. 36341802 10.1016/j.jad.2022.10.040 PMC9613786

[B16] Qian M. Y. Wu G. C. Zhu R. C. (2000). A revised version of Eysenck personality questionnaire short form scale Chinese version (EPQ-RSC). *J. Psychol.* 32 317–323.

[B17] Qiu J. Shen B. Zhao M. Wang Z. Xu Y. A. (2020). Nationwide survey of psychological distress among Chinese people in the COVID-19 epidemic: implications and policy recommendations. *Gen. Psychiatry* 33:100213. 10.1136/gpsych-2020-100213 PMC7061893 32215365

[B18] Su H. Cao J. Zhou Y. Wang L. Xing L. (2018). The mediating effect of coping style on personality and mental health among elderly Chinese empty-nester: a cross-sectional study. *Arch. Gerontol. Geriatr.* 75 197–201. 10.1016/j.archger.2018.01.004 29351838

[B19] Sun L. Sun Z. Wu L. Zhu Z. Zhang F. Shang Z. (2021). Prevalence and risk factors for acute posttraumatic stress disorder during the COVID-19 outbreak. *J. Affect. Disord.* 283 123–129. 33548905 10.1016/j.jad.2021.01.050 PMC7840403

[B20] Sun P. Sun Y. Jiang H. (2019). Gratitude and problem behaviors in adolescents: the mediating roles of positive and negative coping styles. *Front. Psychol.* 10:1547. 10.3389/fpsyg.2019.01547 31379645 PMC6646722

[B21] Tang W. Hu T. Hu B. Jin C. Wang G. Xie C. (2020). Prevalence and correlates of PTSD and depressive symptoms one month after the outbreak of the COVID-19 epidemic in a sample of home-quarantined Chinese university students. *J. Affect. Disord.* 274 1–7. 10.1016/j.jad.2020.05.009 32405111 PMC7217769

[B22] Wang X. Gao L. Shinfuku N. Zhang H. Zhao C. Shen Y. (2000). Longitudinal study of earthquake-related PTSD in a randomly selected community sample in north China. *Am. J. Psychiatry* 157 1260–1266. 10.1176/appi.ajp.157.8.1260 10910788

[B23] Wathelet M. Fovet T. Jousset A. Duhem S. Habran E. Horn M. (2021). Prevalence of and factors associated with post-traumatic stress disorder among French university students 1 month after the COVID-19 lockdown. *Transl. Psychiatry* 11:327. 10.1038/s41398-021-01438-z 34045442 PMC8157529

[B24] WHO (2022). *Coronavirus (COVID-19), 14 October 2022.* Geneva: World Health Organization.

[B25] Wu P. Fang Y. Guan Z. Fan B. Kong J. Yao Z. (2009). The psychological impact of the SARS epidemic on hospital employees in China: exposure. Risk perception, and altruistic acceptance of risk. *Can. J. Psychiatry* 54:302. 10.1177/070674370905400504 19497162 PMC3780353

[B26] Xi Y. Chen R. Yan F. Ma X. Rakofsky J. J. Tang L. (2017). Low post-traumatic stress disorder rate in Chinese in Beijing. China. *Asian J. Psychiatry* 30 79–83. 10.1016/j.ajp.2017.07.003 28837943

[B27] Xie Y. N. (1985). The simplified coping style questionnaire. *Chin. J. Psychol.* 7 123–124.

[B28] Xiong J. Lipsitz O. Nasri F. Lui L. M. Gill H. Phan L. (2020). Impact of COVID-19 pandemic on mental health in the general population: a systematic review. *J. Affect. Disord.* 277 55–64. 32799105 10.1016/j.jad.2020.08.001 PMC7413844

[B29] Xu J. Zheng Y. Wang M. Zhao J. Cheng Y. (2011). Predictors of symptoms of posttraumatic stress in Chinese university students during the 2009 H1N1 influenza pandemic. *Med. Sci. Monit.* 17 60–64. 10.12659/msm.881836 21709644 PMC3539574

[B30] Yang C. X. Fu Y. C. Wang W. (2014). College students social support and coping style: mediator effect of resilience. *Chin. J. Health Psychol.* 22 1065–1067. 10.1111/inm.13081 36300668

[B31] Yin Q. Wu L. Yu X. Liu W. (2019). Neuroticism predicts a long-term PTSD after earthquake trauma: the moderating effects of personality. *Front. Psychiatry* 10:657. 10.3389/fpsyt.2019.00657 31616324 PMC6763688

[B32] Zhou X. Kang L. Sun X. Song H. Mao W. Huang X. (2013). Prevalence and risk factors of post-traumatic stress disorder among adult survivors six months after the Wenchuan earthquake. *Compr. Psychiatry* 54 493–499. 23337407 10.1016/j.comppsych.2012.12.010

